# MicroRNA-214 targets PTK6 to inhibit tumorigenic potential and increase drug sensitivity of prostate cancer cells

**DOI:** 10.1038/s41598-019-46170-3

**Published:** 2019-07-05

**Authors:** Patrice Cagle, Suryakant Niture, Anvesha Srivastava, Malathi Ramalinga, Rasha Aqeel, Leslimar Rios-Colon, Uchechukwu Chimeh, Simeng Suy, Sean P. Collins, Rajvir Dahiya, Deepak Kumar

**Affiliations:** 1 Julius L. Chambers Biomedical Biotechnology Research Institute, North Carolina Central University, Durham, NC 27707 United States; 2 Cancer Research Laboratory, Division of Science and Mathematics, University of the District of Columbia, Washington, DC 20008 United States; 3 Department of Pharmaceutical Sciences, North Carolina Central University, Durham, NC 27707 United States; 4 Department of Radiation Medicine, Georgetown University, Washington, DC 20057 United States; 5 VA Medical Center and University of California San Francisco, San Francisco, CA 94121 United States

**Keywords:** Epithelial-mesenchymal transition, miRNAs

## Abstract

Prostate cancer is the most commonly diagnosed cancer in men with African American men disproportionally suffering from the burden of this disease. Biomarkers that could discriminate indolent from aggressive and drug resistance disease are lacking. MicroRNAs are small non-coding RNAs that affect numerous physiological and pathological processes, including cancer development and have been suggested as biomarkers and therapeutic targets. In the present study, we investigated the role of miR-214 on prostate cancer cell survival/migration/invasion, cell cycle regulation, and apoptosis. miR-214 was differentially expressed between Caucasian and African American prostate cancer cells. Importantly, miR-214 overexpression in prostate cancer cells induced apoptosis, inhibiting cell proliferation and colony forming ability. miR-214 expression in prostate cancer cells also inhibited cell migration and 3D spheroid invasion. Mechanistically, miR-214 inhibited prostate cancer cell proliferation by targeting protein tyrosine kinase 6 (PTK6). Restoration of PTK6 expression attenuated the inhibitory effect of miR-214 on cell proliferation. Moreover, simultaneous inhibition of PTK6 by ibrutinib and miR-214 significantly reduced cell proliferation/survival. Our data indicates that miR-214 could act as a tumor suppressor in prostate cancer and could potentially be utilized as a biomarker and therapeutic target.

## Introduction

Prostate cancer (PCa) is the most common form of cancer and the second leading cause of cancer-related deaths among men in the US, with an estimated 1.3 million cases and 359,000 deaths reported between 2017 and 2018^1,2^. It is the fifth leading cause of cancer-related deaths among men worldwide^1^. Furthermore, African American men are disproportionally affected by this disease and suffer the burden of higher prevalence and mortality rates compared to other ethnic groups^1,3,4^. The heterogeneity of PCa^4^, the lack of effective biomarkers that distinguish potentially aggressive cases from indolent disease, and the significant morbidity associated with treatment interventions are some of the current challenges in this field^5^. Thus identifying novel biomarkers and therapeutic targets are urgently needed to improve PCa diagnosis, prognosis, and treatment.

MicroRNAs, are small single-stranded non-coding RNAs that modulate post-transcriptional gene expression by binding imperfectly to the 3′untranslated regions (3′UTRs) of target messenger RNA (mRNA). This binding regulates both the stability and translation of mRNA. By affecting gene expression, miRNAs can modulate various cellular processes such as development, differentiation, proliferation, survival, cell-cycle control, apoptosis, the stress response, and metabolism^6^. miRNAs themselves are regulated via genetic and epigenetic regulation but can also be dysregulated by alterations in their biogenesis pathway proteins. Altered miRNA expression plays a significant role in diverse cancers, including PCa, and may critically affect its modulation/progression^4,7,8^. Such dysregulation can interfere with cancer hallmarks by either promoting or suppressing tumor development and progression^6,9–11^. Specific cancer hallmarks involving miRNAs that occur during multi-step tumor progression include aberrant growth and proliferation, evasion of growth suppressors, resistance to cell death, metabolic reprogramming, host microenvironment interactions, induced angiogenesis, regulation of drug resistance, immune modulation, and invasion and metastasis to distant organs^4,12–15^. MicroRNAs also serve as biomarkers of disease aggressiveness and therapeutic resistance or as therapeutic targets of pharmacological or oligonucleotide interventions^4,7,8,16–20^.

We previously demonstrated that expression of miR-214 is downregulated in prostate tumors compared with adjacent normal tissues and urine^19^. miR-214 is known to modulate numerous cancer-related signaling cascades in multiple cancers^21–23^. miR-214 is located in intron 14 of the Dynamin-3 gene on chromosome 1q24.3. Previous studies suggest that miR-214 could have both tumor suppressor and oncogenic functions in various types of cancer, including melanoma, ovarian, and breast cancer^21^. In cervical cancer, miR-214 acts as a tumor suppressor that inhibits cell proliferation/migration/invasion and increases drug sensitivity by down-regulating MEK3, JNK1, FOXM1, Bcl2, and HMGA1^24–27^. In gliomas and brain tissue, miR-214 similarly inhibits cell proliferation and migration by regulating Caspase 1-mediated pyroptosis^28^. In breast cancer, miR-214 overexpression induces both oncogenic activity by targeting the PTEN-PI3K/AKT signaling pathway^29^ and tumor suppressor activity by regulating the RFWD2-p53 cascade^30^, inhibiting cell proliferation and migration, and promoting apoptosis^31^. Thus, the role of miR-214 is likely context and cell type specific, with its diverse functions depending on its specific target genes. The exact function of miR-214 in PCa remains to be elucidated.

Protein tyrosine kinase 6 (PTK6), also called breast tumor kinase (BRK/PTK6), is a non-receptor intracellular tyrosine kinase that is expressed in various normal epithelia, including the linings of the gastrointestinal tract, skin, oral cavity, and prostate. In these regions, PTK6 expression is anti-oncogenic in signaling pathways that control cell survival, cell cycle, and differentiation^32–34^. PTK6 was first identified in cultured human melanocytes^35^, breast tumor cells^36^, and the murine gastrointestinal tract^37^. Increased PTK6 expression is found in PCa, especially at metastatic stages, and in other cancer types such as lung, bladder, ovarian, cervical, pancreas, gastric, head and neck cancers, and B- and T-cell lymphomas^32,38^. Metastatic human prostate tissue samples also express higher levels of PTK6 mRNA relative to normal tissues and primary tumor tissue samples^39^. Increased PTK6 expression promotes pro-oncogenic phenotypes such as cell proliferation, cell cycle, cell migration, angiogenesis, apoptosis, and autophagy^33,38^. While PTK6 is found in the nucleus of normal prostate epithelial cells, it relocates to the cytoplasm and cell membrane in PCa cells^32,33,40^. Knockdown of cytoplasmic PTK6 in prostate cancer PC3 cells limits cell proliferation, migration, and anoikis resistance^33,39^. Alternatively, activation of membranous PTK6 promotes epithelial-mesenchymal transition (EMT) by activating AKT^41^.

In the current study, we explore the role of miR-214 in PCa tumorigenesis and its potential as a biomarker and therapeutic target. We demonstrate that miR-214 inhibits cell proliferation, migration, and invasion in PC3, DU145, and MDA-PCa-2b PCa cells. We also showed that miR-214 overexpression inhibits cell proliferation and colony formation inducing G_0_/G_1_ and G_2_/M cell cycle arrest and apoptosis. miR-214 exerts its action by directly targeting the 3′UTR region of PTK6 to reduce its expression. PTK6 overexpression significantly reduced the inhibitory effects of miR-214 on cell proliferation. Moreover, PTK6 inhibition by miR-214 or the co-treatment of miR-214 and BTK inhibitor ibrutinib significantly decreased cell proliferation in PCa cells.

## Results

### miR-214 inhibits prostate cancer cell viability and colony formation

**Figure 1:**
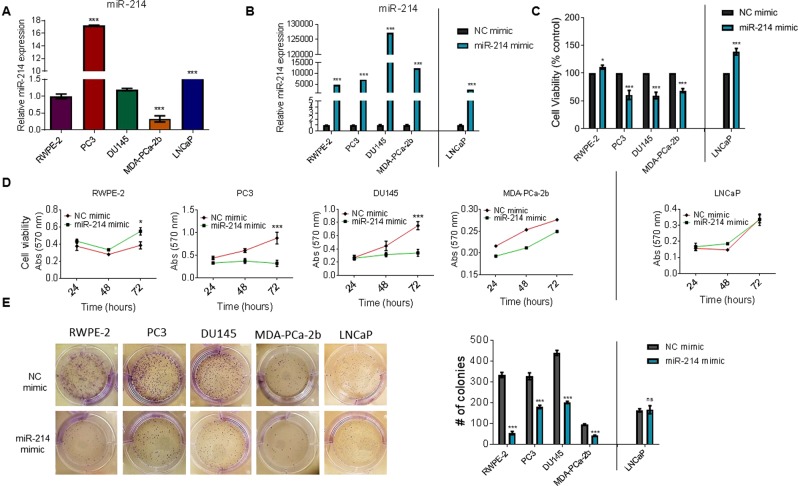
Effects of miR-214 on cell proliferation and colony formation in prostate cancer cells. (**A)** RWPE-2, PC3, DU145, MDA-PCa-2b, and LNCaP cells were cultured for 30 h, and the relative endogenous expression of miR-214 was analyzed by RT/qPCR. **(B)** RWPE-2, PC3, DU145, MDA-PCa-2b cells, and LNCaP were transfected with negative control (NC) mimic miRNA or miR-214 mimic for 48 h. miR-214 expression levels were then determined by RT/qPCR. (**C**) RWPE-2, PC3, DU145, MDA-PCa-2b, and LNCaP cells were transfected with NC or miR-214 mimic. After 48 h, the effect of the miR-214 expression on cell viability was determined by MTT assay. (**D**) The time-dependent effects of miR-214 expression on cell viability were determined by MTT assay. (**E**) The effect of NC or miR-214 mimic on cell colony formation was determined by colony formation assay (left). The number of colonies were measured by ImageJ software. The data represent the colony count ± SEM (right). One-way and two-way ANOVA followed by Tukey’s multiple comparisons test were used to determine statistical significance for RT/qPCR and MTT assay, respectively. Results are representative of three independent experiments. *P < 0.05, **P < 0.005, ***P < 0.0005 compared with NC-transfected cells.

To determine the biological functions of miR-214 in prostate cells, we first used RT/qPCR to quantify miR-214 expression in the transformed-immortalized human prostate cell line (RWPE-2) and different PCa cell lines (PC3, DU145, MDA-PCa-2b, and LNCaP; Fig. 1A). miR-214 was differentially expressed in PCa cells. Specifically, African-American MDA-PCa-2b PCa cells expressed significantly lower miR-214 than Caucasian PCa cells PC3, DU145, and LNCaP (Fig. 1A). To explore the potential role of miR-214 in the PCa progression, RWPE-2, PC3, DU145, MDA-PCa-2b, and LNCaP cells were transiently transfected with miR-214 or NC (negative control) mimics, and the expression levels of miR-214 were measured by RT/qPCR. We observed that miR-214 was significantly increased in the miR-214 mimic group compared to NC mimic (Fig. 1B), confirming the miRNA mimic transfection efficiency. The results of a cell proliferation/viability assay 48 h post-transfection revealed that miR-214 overexpression dramatically suppressed the proliferation and viability of PC3, DU145, and MDA-PCa-2b cells compared with the NC mimic group, while proliferation was significantly increased in the LNCaP and RWPE-2 cells (Fig. 1C). Similarly, time-dependent miR-214 overexpression (24 to 72 h) inhibited cell proliferation in PC3, DU145, and MDA-PCa-2b cells compared with NC-transfected cells, but increased cell proliferation in RWPE-2 cells and did not affect LNCaP cells at 72 h. (Fig. 1D). We also examined how miR-214 overexpression affected cell colony formation (Fig. 1E, left and right panels). In RWPE-2, PC3, DU145, and MDA-PCa-2b cells, miR-214 overexpression limited cell colony formation (83.9%, 45.1%, 54.2%, and 56.9%, respectively) over a period of 5–7 days compared with NC-transfected cells (Fig. 1E, left and right panels). Interestingly, no significant effects of miR-214 on the clonogenic potential of LNCaP cells were observed (Fig. 1E, right panel). Collectively, these data suggested that miR-214 inhibits cell proliferation and clonogenic potential in PC3, DU145, and MDA-PCa-2b PCa cells, but not in RWPE-2 and LNCaP cells.

### miR-214 induces G_0_/G_1_ and G_2_/M cell cycle arrest and increases cell apoptosis in prostate cancer cells

**Figure 2:**
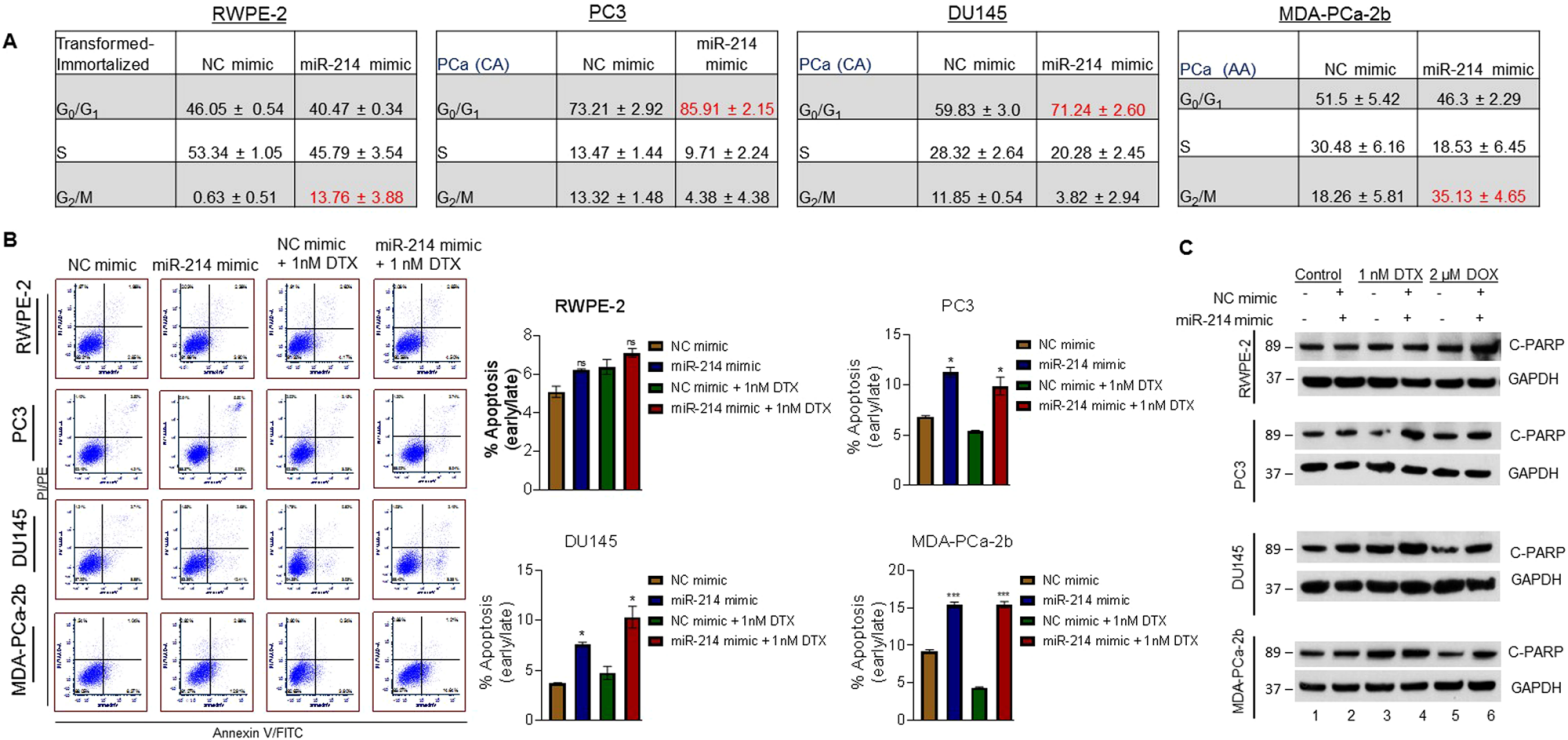
Effects of miR-214 overexpression on cell cycle progression and apoptosis. **(A)** RWPE-2, PC3, DU145, and MDA-PCa-2b cells were transfected with NC or miR-214 mimic. The percentages of cells in the G_0_/G_1_, S, and G_2_/M cell cycle phases for each cell line are shown. The red numbers indicate a significant increase in cell cycle phase in the miR-214-transfected cells compared to NC-transfected cells. **B)** RWPE-2, PC3, DU145, and MDA-PCa-2b cells were transfected with NC or miR-214 mimic with and without 1 nM docetaxel (DTX). Flow cytometry was used to determine the percentages of Annexin V-FITC+ cells (right lower quadrant, early apoptosis) and Annexin V-FITC+/PI+ cells (right upper quadrant, late apoptosis). Representative plots (left) and the corresponding quantification (right) are shown. **C)** RWPE-2, PC3, DU145, and MDA-PCa-2b cells were transfected with NC or miR-214 mimic. Cells were also treated with DTX or doxorubicin (DOX). The effects of miR-214 mimic alone or in combination with the drugs above on C-PARP and GAPDH expression were analyzed by western blotting. One-way ANOVA with Tukey’s multiple comparisons test was used to determine statistical significance. The data represent mean ± SEM and results are representative of three independent experiments *P < 0.05, ***P < 0.0005.

We next examined the molecular mechanisms by which miR-214 modulated cell growth/proliferation. First, we determined how miR-214 overexpression affected cell cycle progression in PCa cells using propidium iodide (PI) staining. Overexpression of miR-214 in RWPE-2, PC3, DU145, and MDA-PCa-2b cells, significantly altered the cell cycle phases, particularly G_0_/G_1_ and G_2_/M, compared with NC-transfected cells (Fig. 2A). Specifically, miR-214 overexpression significantly increased the percentage of cells in G_0_/G_1_ (PC3, 12.7%; DU145, 11.41%) and G_2_/M (RWPE-2, 13.13%; MDA-PCa-2b, 16.87%) compared with NC-transfected cells. These results indicated that miR-214 overexpression prevented the G_1_-to-S phase transition in PC3 and DU145 cells, thus inhibiting cell proliferation through G_0_/G_1_ cell cycle arrest. We also observed an increase in G_2_/M phase in MDA-PCa-2b and RWPE-2 cells suggesting cell-specific cell cycle regulatory mechanisms (Fig. 2A).

Because miR-214 inhibited cell proliferation and induced cell cycle arrest, we further analyzed the effect of miR-214 overexpression on cell apoptosis (Fig. 2B, left and right panels). Fluorescence-activated cell sorting (FACS) analysis demonstrated that miR-214 overexpression significantly increased the percentage of apoptotic (Annexin V+ ) PC3 (4.4%), DU145 (3.88%), and MDA-PCa-2b (6.23%) cells compared with NC-transfected cells. However, this effect was not observed in RWPE-2 cells (Fig. 2B, left and right panels). Furthermore, miR-214-transfected cells when treated with the anti-cancer drug, Docetaxel (DTX), significantly increased the percentage of apoptotic cells compared with NC-transfected and DTX-treated PCa cells (Fig. 2B, left and right panels). Western blotting results showed increased expression of cleaved PARP (c-PARP) in the miR-214-transfected PC3, DU145, and MDA-PCa-2b cells compared to the NC-transfected cells (Fig. 2C, lanes 1 & 2). Combining miR-214 overexpression and treatment with DTX or doxorubicin (DOX, cell apoptosis-inducing reagent) also increased c-PARP expression compared with NC-transfected and DTX- or DOX-treated PC3, DU145, and MDA-PCa-2b cells (Fig. 2C, Lanes 3–6). These data strongly suggested that miR-214 regulates the cell cycle and induces apoptosis in PCa cells.

### miR-214 inhibits cell migration and invasion by regulating EMT markers

**Figure 3:**
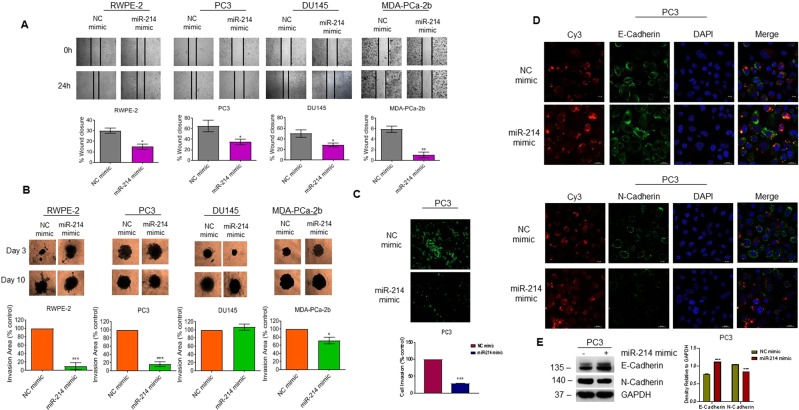
Effects of miR-214 overexpression on prostate cancer cell migration and invasion. (**A**) Wound healing assay: RWPE-2, PC3, DU145, and MDA-PCa-2b cells were transfected with NC or miR-214 mimic for 48 h. The effect of the miR-214 expression on cell migration was analyzed using the wound healing assay. Representative images of the wound healing assay (top) and the calculated scratch area (bottom) are shown. **(B)** 3D spheroid invasion assay: RWPE-2, PC3, DU145, and MDA-PCa-2b cells were transfected with NC or miR-214 mimic and then analyzed using the 3D spheroid assay. 3D spheroid images (top) and the quantified 3D invasion area (bottom) were determined by subtracting pre-invasion (day 3) from post-invasion (day 10) area. (**C**) Trans-well invasion assay: PC3 cells were transfected with NC or miR-214 mimic. After 48 h, the effect of miR-214 mimic on cell invasion was analyzed by trans-well invasion assay in miR-214-transfected PC3 cells stained with Calcein-AM (Green fluorescence, top) and quantified (bottom). The invasion assay was performed in duplicate, and results are representative of three independent experiments. **(D**,**E)** The effects of NC or miR-214 mimic transfection on the expression of E-Cadherin (top, green) and N-Cadherin (bottom, green) in PC3 cells were analyzed by (**D**) immunofluorescence and (**E**) western blotting. *P < 0.05, **P < 0.005, ***P < 0.0005 compared with NC-transfected cells.

We evaluated the effects of miR-214 overexpression on PCa cell migration and invasion using wound healing migration, matrigel invasion, and 3D spheroid formation assays. The wound healing migration assay revealed that overexpressing miR-214 significantly inhibited cell migration and wound closure in RWPE-2 (50.4%), PC3 (46.2%), DU145 (42.7%), and MDA-PCa-2b cells (82.5%) (Fig. 3A, upper and lower panels). To elucidate the effects of miR-214 on PCa cell invasion, we next compared cells transfected with NC or miR-214 mimic for their ability to form spheroids in 3D culture and invade the surrounding extracellular matrix (ECM). miR-214 overexpression dramatically reduced 3D cell invasion in RWPE-2 (90%), PC3 (83.3%) and MDA-PCa-2b (28%) cells compared with NC-transfected cells; however, miR-214 did not affect DU145 3D cell invasion (Fig. 3B, upper and lower panels). We also evaluated the effects of miR-214 on the invasion of PC3 cells using a trans-well invasion assay (Fig. 3C). This assay clearly demonstrated that miR-214 overexpression significantly decreased PC3 cell invasion (71.3%) compared with NC-transfected cells (Fig. 3C, upper and lower panels).

EMT markers also play important roles in cell migration and invasion and decreased levels of E-cadherin (EMT Marker) are associated with high prostate tumor grade and poor prognosis^41^. Zheng *et al*. showed that high levels of PTK6 predict poor prognosis in patients with PCa and PTK6 levels are inversely correlated with E-Cadherin expression in metastatic PCa^41^. Because we observed that miR-214 overexpression decreased PC3 cell migration and invasion, we also evaluated how miR-214 overexpression affected the expression of the EMT markers, E-Cadherin and N-Cadherin, using immunofluorescence and immunoblotting (Fig. 3D,E). Our immunofluorescence data suggested that miR-214 overexpression in PC3 cells significantly increased E-Cadherin expression, while slightly decreasing N-Cadherin relative to NC-transfected cells (Fig. 3D, upper and lower panels). Immunoblotting data confirmed this effect (Fig. 3E, left and right panels). These data suggested that miR-214 inhibits cell migration and invasion by regulating EMT markers in PCa cells.

### miR-214 targets the 3′UTR region of PTK6 and inhibits PTK6 expression

**Figure 4:**
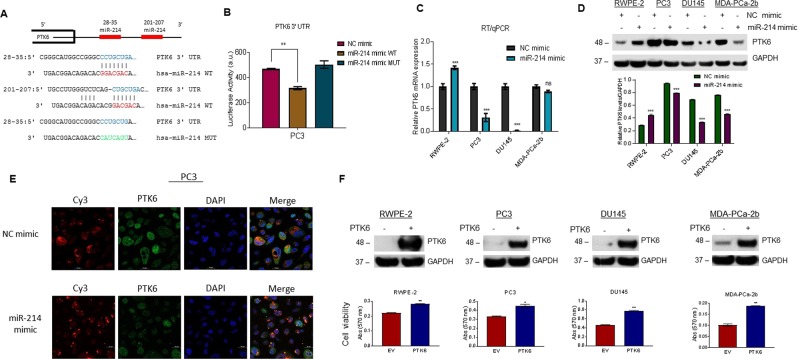
miR-214 targets PTK6 and inhibits its expression. (**A)** The binding sites of miR-214 in the 3′UTR region of PTK6 were analyzed by TargetScan (http://www.targetscan.org/vert_72/) and presented (top). We mutated the binding nucleotides of miR-214 to the 3′UTR region of PTK6 to generate a mutant miR-214 mimic. Constructs of the wild-type and mutant miR-214 mimics are shown (bottom). **(B)** Luciferase reporter assay: PC3 cells were first transfected with one µg of PTK6-3′UTR-Luciferase reporter construct for 18 h and then transfected with NC, wild-type miR-214, or mutant miR-214 mimic for an additional 24 h. Transfected cells were lysed, and luciferase activity was then measured. **P < 0.005 compared with NC or wild-type miR-214 mimic. (**C**) RWPE-2, PC3, DU145, and MDA-PCa-2b cells were transfected with NC or miR-214 mimic. After 48 h, relative *PTK6* expression was analyzed by RT/qPCR. ***P < 0.0005 compared with NC-transfected cells. **(D)** RWPE-2, PC3, DU145, and MDA-PCa-2b cells were transfected with NC or miR-214 mimic. After 48 h, PTK6 protein levels were analyzed by western blotting (top) and quantified using ImageJ (bottom). ***P < 0.0005 compared with NC-transfected cells**. (E)** PC3 cells were transfected with NC or miR-214 mimic for 48 h, and then PTK6 expression (green) was visualized by immunofluorescence as described in the materials and methods section. (**F**) RWPE-2, PC3, DU145, and MDA-PCa-2b cells were transfected with PTK6 plasmid or empty vector plasmid, and expression of PTK6 protein levels were analyzed by western blotting (upper panel). Effect of PTK6 overexpression on cell viability of prostate cancer cells was measured by MTT assay and plotted (lower panel). The data represent mean ± SEM. One-way ANOVA with Tukey’s multiple comparisons test was used to determine statistical significance for RT/qPCR and western blot analysis.

Because miRNA-214 targets numerous oncogenes and tumor suppressor genes involved in human cancer, we used TargetScan, an online software tool (http://www.targetscan.org/vert_72/: accessed May 10, 2018), to predict possible miR-214-binding targets. The TargetScan analysis revealed that miR-214 potentially binds two regions of the 3′UTR (28–35 and 201–207) of PTK6 mRNA (Fig. 4A). To determine whether miR-214 binds directly with the PTK6–3′UTR, we co-transfected PC3 cells with miR-214, NC, or miR-214-mutated-oligonucleotide mimics and a light-switch reporter plasmid that contained a region of full-length 3′UTR of *PTK6*, which harbors the miR-214 target site. We then performed a reporter luciferase assay, which clearly showed that the wild-type miR-214 mimic significantly decreased (32.5%) luciferase activity relative to the NC mimic (Fig. 4B). Alternatively, the mutant miR-214 mimic was unable to bind with the PTK6-3′UTR and suppress luciferase activity compared with wild-type miR-214-transfected cells, suggesting that miR-214 targets the PTK6-3′UTR (Fig. 4B). We also examined how miR-214 mimic transfection affected *PTK6* mRNA levels using RT/qPCR (Fig. 4C) and PTK6 protein levels by immunoblotting (Fig. 4D). RT/qPCR and immunoblotting data both showed that miR-214 transfection downregulated *PTK6* mRNA and protein expression in PC3, DU145, and MDA-PCa-2b cells compared with NC-transfected cells; however, miR-214 transfection did not suppress *PTK6* mRNA and protein levels in RWPE-2 cells (Fig. 4C,D). Immunofluorescence analysis of PC3 cells also showed that miR-214 transfection decreased PTK6 expression relative to NC-transfected cells (Fig. 4E). Thus, miR-214 negatively regulates PTK6 protein expression by targeting the PTK6-3′UTR.

Since miR-214 negatively regulates endogenous PTK6 protein, we next overexpressed PTK6 and examined the association between ectopic PTK6 expression on cell viability/proliferation in PCa cells. Immunoblotting data demonstrated overexpression of PTK6 protein in all indicated cells lines compared to empty vector transfected cells (Fig. 4F, upper panel). We also measured the effect of overexpression of PKT6 on cell survival (Fig. 4F, lower panel). PTK6-overexpression increased cell survival in RWPE-2 (26.5%), PC3 (34.2%) & DU145 (67.8%), and MDA-PCa-2b (85.3%) cells compared with empty vector transfected cells (Fig. 4F, lower panel) suggesting that, PKT6 expression positively modulates cell survival in PCa cells.

### miR-214 downregulates PTK6 and inhibits cell proliferation and cell colony formation in prostate cancer cells

**Figure 5:**
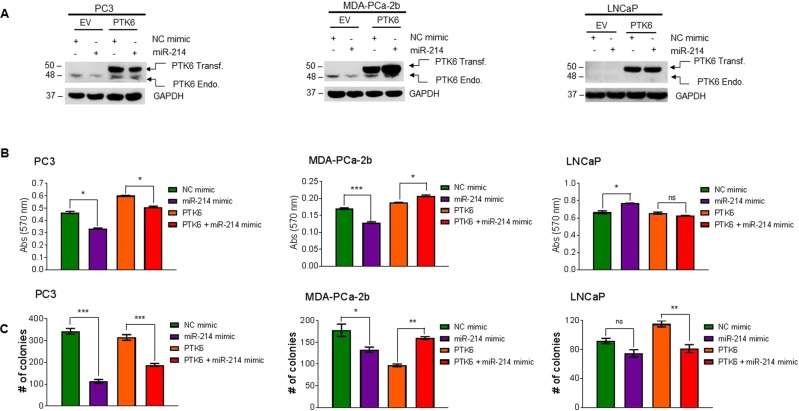
Effects of PTK6 overexpression on prostate cancer cell proliferation and colony formation. (**A)** Western blot analysis: PC3, MDA-PCa-2b, and LNCaP cells were co-transfected with empty vector (EV) or PTK6 plasmids as indicated for the first 24 h and then transfected with NC or miR-214 mimic for an additional 24 h. Cell lysates (40 µg) were immunoblotted with anti-PTK6 and anti-GAPDH antibodies. (**B**) MTT assay: PC3, MDA-PCa-2b, and LNCaP cells were transfected as indicated in (**A**) and after 48 h, cell proliferation was analyzed by MTT assay. (**C**) Cell colony formation assay: PC3, MDA-PCa-2b, and LNCaP cells were transfected with EV or PTK6 plasmids for 24 h and then transfected NC or miR-214 mimic for an additional 24 h. Cells were then trypsinized, counted, and re-plated in 6-well plates for analysis using colony formation assays. The data represent mean ± SEM. One-way ANOVA followed by Tukey’s multiple comparisons test was used to determine statistical significance for the cell viability and colony formation assays. Results are representative of three independent experiments. *P < 0.05, **P < 0.005, ***P < 0.0005 compared with NC mimic or PTK6 transfected cells.

PTK6 induces oncogenic signaling in PCa^33,38,42^, and our data suggested that PTK6 increases cell viability/proliferation. Since miR-214 targets PTK6 expression, we further investigated whether PTK6 overexpression could attenuate the effects of miR-214 on PCa cell survival. To test this, we co-transfected PC3, MDA-PCa-2b, and LNCaP cells with empty vector or PTK6 cDNA plasmid and NC mimic or miR-214 mimic and cell lysates were then immunoblotted (Fig. 5A). As expected, PTK6 transfection increased exogenous PTK6 levels compared with empty vector-transfected cells in all three cell lines. PTK6 and miR-214 co-transfection decreased the levels of endogenous PTK6 protein in PC3 and MDA-PCa-2b cells compared with PTK6 and NC mimic transfected cells, and, miR-214 mimic transfection did not affect the exogenous expression of PTK6 protein in MDA-PCa-2b cells (Fig. 5A, left two panels). Under similar experimental conditions, the effect of miR-214 and PTK6 on cell survival and cell colony formation were analyzed (Fig. 5B,C left two panels). As expected, miR-214 overexpression alone inhibited cell viability in PC3 (28.0%) and MDA-PCa-2b (24.1%) cells. Interestingly, co-transfecting with miR-214 in PTK6 overexpressing cells decreased and increased cell viability/survival in PC3 and MDA-PCa-2b cells respectively (Fig. 5B, left two panels) compared to the NC mimic and PTK6 overexpressing cells. These results suggested that although miR-214 inhibited the expression of endogenous PTK6, cDNA-mediated expression of PTK6 (exogenous) restored cell survival in MDA-PCa-2b cells (Fig. 5B, middle panel). Similarly, miR-214 overexpression inhibited colony formation in PC3 (66.9%) and MDA-PCa-2b (25.3%) cells, while combined PTK6 and miR-214 overexpression restored cell clonogenicity significantly in MDA-PCa-2b cells, but not in PC3 cells (Fig. 5C, left two panels). However, in LNCaP cells, which expressed lower/undetectable levels of endogenous PTK6 compared with PC3 and MDA-PCa-2b cells (Fig. 5A right upper panel), overexpressing miR-214 alone or miR-214 and PTK6 did not significantly inhibit cell survival, but decreased cell colony formation (Fig. 5B,C, right panels). Altogether, these results demonstrated that miR-214 inhibits cell proliferation and cell growth by downregulating PTK6 in PCa cells.

### miR-214 sensitizes prostate cancer cells to ibrutinib by targeting PTK6

**Figure 6:**
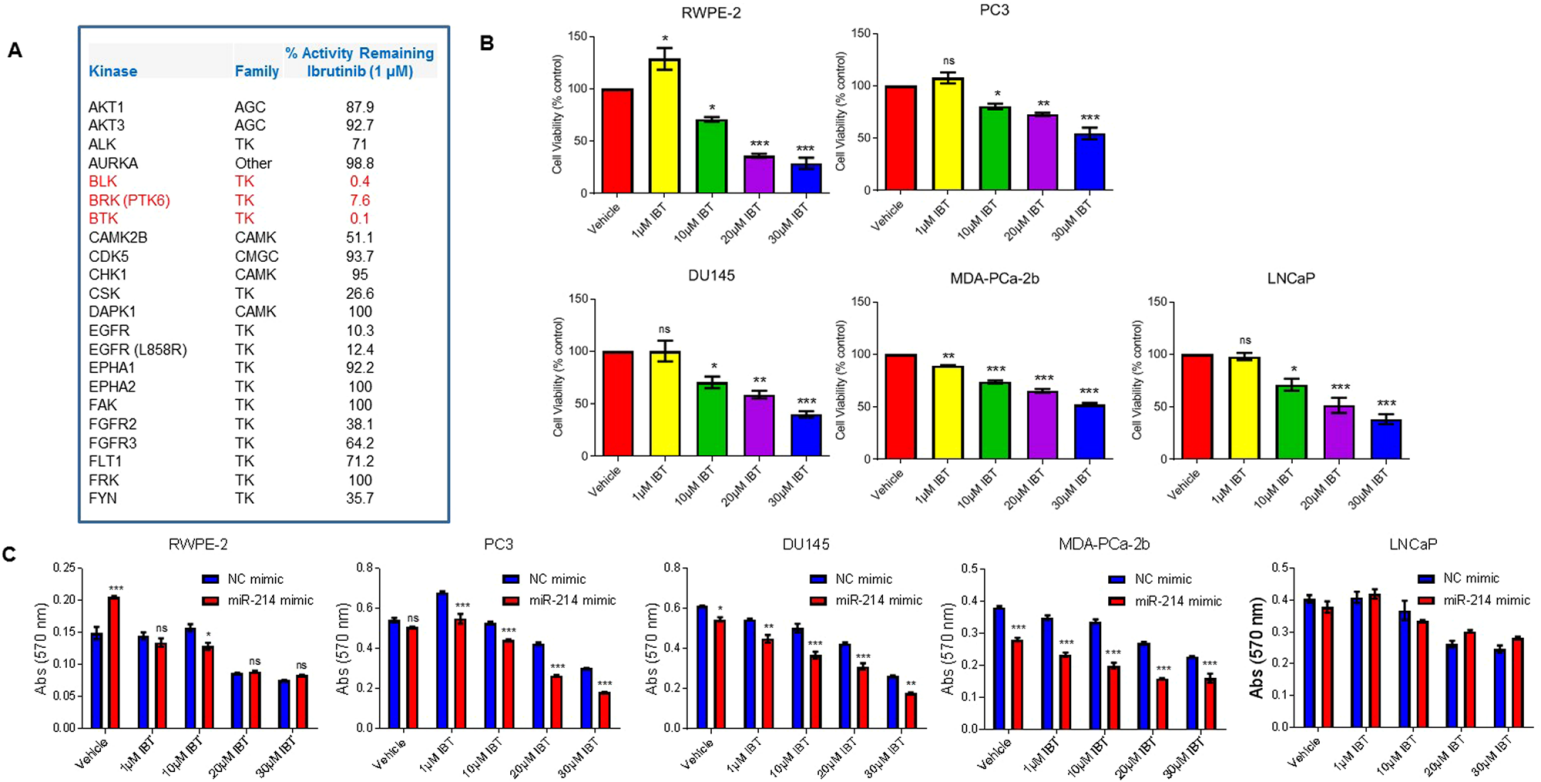
Effects of ibrutinib on prostate cancer cell proliferation. (**A**) Ibrutinib inactivates PTK6 activity *in vitro* (as indicated in red). (**B**) RWPE-2, PC3, DU145, MDA-PCa-2b, and LNCaP cells were treated with increasing concentrations of ibrutinib for 48 h, and then cell proliferation was measured by MTT assay. *P < 0.05, **P < 0.005, ***P < 0.0005 compared with vehicle-treated cells. (**C**) RWPE-2, PC3, DU145, MDA-PCa-2b, and LNCaP cells were transfected with NC or miR-214 mimic for 24 h and then treated with increasing concentrations of ibrutinib for an additional 24 h. Cell survival was measured by MTT assay. *P < 0.05, **P < 0.005, ***P < 0.0005 compared with NC/miR-214-transfected and ibrutinib-treated cells. One-way ANOVA and two-way ANOVA, followed by Tukey’s multiple comparisons test were used to determine statistical significance. Results are representative of three independent experiments.

Ibrutinib (IBT) is an irreversible inhibitor of Bruton’s Tyrosine Kinase (BTK) that was initially developed and approved to treat B-cell malignancies such as Mantle Cell Lymphoma (MCL) and Chronic Lymphocytic Leukemia (CLL)^43–45^. It is currently used in the phase II clinical trial to treat PCa (NCT02643667). IBT was previously shown to inhibit cell proliferation and induce apoptosis by targeting BTK in PCa cells^46^. We tested the effect of IBT on kinase inhibition using an *in vitro* kinase assay as described previously^47^. The data demonstrated that IBT selectively inactivates 92% of PTK6 (BRK) activity and also inactivates more than 99% activity of other Src kinases such as BTK and BLK (BLK proto-oncogene, Src family tyrosine kinase) (Fig. 6A). To test the effect of IBT on PCa cell growth, we used our four PCa cell lines and transformed-immortalized prostate cells (RWPE-2). Dose-dependent treatment of RWPE-2, PC3, DU145, MDA-PCa-2b, and LNCaP cells with IBT significantly reduced cell viability (30% to 70%) compared with vehicle-treated cells, suggesting that inhibiting endogenous PTK6 with IBT may trigger cell death in prostate cells (Fig. 6B).

To assess whether miR-214 affected IBT sensitivity in PCa cells, we then transiently transfected these cells with miR-214 and treated them with varying concentrations of IBT (1–30 µM) for 48 h. Compared to the NC mimic, miR-214 overexpression significantly reduced the viability of PC3, DU145, and MDA-PCa-2b cells by 37.7%, 26.6%, and 40.6%, respectively, at a concentration of 20 µM IBT (Fig. 6C). IBT significantly decreased cell viability in LNCaP cells (Fig. 6B) but we did not observe any effect of miR-214 overexpression on IBT sensitivity in LNCaP cells (Fig. 6C). This may be due to LNCaP cells expressing undetectable/lower PTK6 levels compared with PC3, DU145, or MDA-PCa-2b cells. Collectively the data suggested that miR-214 sensitizes PCa cells to IBT by targeting PTK6.

## Discussion

Proliferation, migration, and invasion are prominent biological characteristics of tumor progression, contributing to metastasis, drug resistance, and high mortality rates in PCa. Despite recent therapeutic advances, there is still a lack of discriminatory biomarkers and effective drug regimens, decreasing survival^4^. There is an unmet need for more sensitive and specific biomarkers in addition to PSA. Recent studies have highlighted the multifunctional roles of miRNAs in the risk, development, and progression of many cancers^48–53^, including PCa^8^. In the present study, we investigated the biological functions of miR-214 in PCa cells. Our data indicates that miR-214 is differentially expressed between Caucasian and African-American PCa cells and the lower expression of miR-214 could contribute to the aggressiveness associated with the health disparity in African American patients with prostate cancer. African American men have disproportionately high incidence and mortality rates of prostate cancer when compared to Caucasian men^5,54,55^. The identification of molecular factors/mechanisms that contribute to this health disparity could improve diagnosis and therapeutic outcomes. miR-214 overexpression resulted in cell cycle arrest, decreased cell viability, and clonogenicity in PC3, DU145, and MDA-PCa-2b PCa cells. We also observed inhibition of epithelial-mesenchymal transition (EMT) related processes such as migration, and 3D invasion along with modulation of EMT markers. Our data corroborates with previous studies that have shown that miR-214 overexpression plays a role in tumor cell proliferation, growth and invasion, and tumor progression and metastasis^22,25,27,28,31,56^.

miR-214 modulates cancer development and progression by targeting different target genes and signaling pathways^21^. Here, we show that miR-214 targets and binds the 3′UTR region of protein tyrosine kinase 6 (PTK6), thereby reducing PTK6 expression. PTK6 is known to regulate cell survival, differentiation, and metastasis. Increased expression and an oncogenic role of PTK6 has been shown in multiple cancers including PCa^33,34,57,58^. In PCa, PTK6 overexpression is associated with cell growth, metastasis, and poor prognosis^33,39,41,59^. Differences in PTK6 intracellular localization have been observed in prostate tumor cells. Specifically, PTK6 localizes to the nucleus of normal prostate epithelial cells but relocates to the cytoplasm and cell membrane in PCa cells^32,33,40^. Knockdown of cytoplasmic PTK6 in PC3 cells decreases cell proliferation and migration^33,39^, while activation of membranous PTK6 promotes EMT by activating AKT^41^. Higher expression of PTK6, its altered localization, and activity in PCa indicates that it may be considered a therapeutic target^32,40^. Activation of PTK6 has been shown to promote invasive PCa induced by the loss the PTEN (Phosphatase and tensin homolog)^34^. In this study, we demonstrate that PTK6 is targeted by miR-214, a tumor suppressor microRNA. We previously demonstrated that miR-214 is downregulated in PCa tissues when compared with benign adjacent tissue^19^. A tumor suppressor role of miR-214 has been demonstrated in multiple cancers such as gliomas, bladder, colorectal, esophageal squamous cell carcinoma, and hepatocellular carcinoma^21,22,25,27,28,31,56,60–62^. miR-214 modulates drug sensitivity in breast and cervical cancer cells^24,26,30,63^. Using cell cycle analysis and cell proliferation, colony formation, and invasion assays, we confirmed that miR-214 overexpression suppressed PCa cell progression by downregulating PTK6. We must note that miR-214 may target several oncogenic molecules such as BTK and PTK6 (this study) and may exert its effect by targeting multiple genes.

Ibrutinib (IBT), is a therapeutic drug approved primarily for liquid malignancies that targets Bruton Tyrosine Kinase (BTK). BTK is aberrantly expressed in PCa tissues and cells and promotes PCa cell growth^64^. In this study, we also demonstrate that IBT targets PTK6 which as described earlier is also a potential target in PCa. IBT also targets Fibroblast Growth Factor Receptor 2 (FGFR2)^65,66^ and mutant- Epidermal Growth Factor Receptor (mut-EGFR)^67,68^ in cancer cells. In addition to PTK6, we also observed modest inactivity of FYN, another Src kinase, which plays an important role in cancer as well as diseases of the central nervous system^69,70^. Through this study, we have added PTK6 to the list as a new target for IBT. IBT approved as a treatment in patients with B cell malignancies, is associated with high response rates in patients with relapsed CLL and MCL^43–45^. IBT may also be of benefit to patients with PCa since it is known to target BTK and as shown here to target PTK6, both of which are highly expressed and promote tumorigenesis in PCa.

In conclusion, we demonstrate that miR-214 decreased cell proliferation, migration, and invasion, by repressing PTK6 expression and activity. Together, our data suggest a possible role for miR-214 regulation of gene expression in PCa progression and miR-214 could be explored as a potential therapeutic target in PCa.

## Materials and Methods

### Cell culture

Cancer cell lines were obtained from the Georgetown University Lombardi Comprehensive Cancer Center cell culture repository. Prostate cancer PC3, DU145, and LNCaP cells (Caucasian) were grown in Roswell Park Memorial Institute (RPMI) medium (Invitrogen, Carlsbad, CA) containing 5% fetal bovine serum (FBS; Access Biologicals, Vista, CA), 2 mM glutamine, and 25 µg/ml gentamicin (Invitrogen). RWPE-2 transformed-immortalized human prostate cells were cultured in keratinocyte serum-free medium (K-SFM) supplemented with 0.05 mg/ml bovine pituitary extract, 5 ng/ml epidermal growth factor, and 1% penicillin/streptomycin. African-American PCa MDA-PCa-2b cells were cultured in human prostate cell 1 (HPC1) medium supplemented with 10% FBS (AthenaES, Baltimore, MD). All cell lines were maintained at 37 °C in a humidified atmosphere containing 5% CO_2_, grown for at least 24 h, and used for experiments once they reached 70–80% confluence. To facilitate MDA-PCa-2b attachment, FNC Coating Mix (AthenaES) was implemented for all cell culture plates and dishes.

### miR-214 and Negative Control (NC) mimic transfection

RWPE-2, PC3, DU145, MDA-PCa-2b, and LNCaP cells (2–5 × 10^5^) were transiently transfected with 50 nM mirVana miR-214 mimic (MC12124; Life Technologies Corporation, Carlsbad, CA) or NC mimic (Life Technologies Corporation). To confirm the transfection efficiency, PC3 cells were transfected with custom Cy3-labeled NC or miR-214 mimic (Bioneer, Oakland, CA). The cells were transfected using Lipofectamine 2000 reagent (Life Technologies Corporation) following the manufacturer’s protocol.

### MTT assay

Cell survival/proliferation was performed using 3-(4,5-dimethylthiazol-2-yl)-2,5-diphenyltetrazolium bromide (MTT) reagent (MP Biochemicals, Santa Ana, CA). Cells (2–5 × 10^5^) were seeded onto 6-well plates overnight and then transfected with 50 nM NC or miR-214 mimic (or 2 µg EV or PTK6) for 24 h. The transfected cells were then harvested and seeded onto 96-well plates (2.5–5 × 10^3^ cells/well) and grown for 24, 48, or 72 h. For ibrutinib (IBT) experiments, transfected cells were seeded onto 96-well plates and treated with the indicated concentrations of IBT for 48 h. Following exposure to specific transfection/treatments, cells were incubated with 10 µl/well of MTT reagent (5 mg/ml) for 2 h at 37 °C in a cell culture incubator. Formazan crystals were dissolved in dimethyl sulfoxide (DMSO), and cell survival/proliferation was quantified by reading the plates at 570 nm using a Fluostar Omega plate reader (BMG Lab Tech, Cary, NC).

### Colony formation assays

For colony formation assays, RWPE-2 and PCa cells (2–5 × 10^5^) were transfected with NC or miR-214 mimic. After 24 h, transfected cells were trypsinized, counted, and seeded onto 6-well plates at a density of 2,000–5,000 cells/well and cultured for 7–9 days. For PTK6 overexpression experiments, cells (2–5 × 10^5^) were seeded onto 6-well plates and then transfected with PTK6 cDNA plasmid (0.5 µg) or empty vector plasmid. After 24 h, cells were transfected with 50 nM miR-214 or NC mimic for an additional 24 h. Next, cells were trypsinized, counted, and plated at a density of 2,000–5,000 cells/well and cultured for 7–9 days in 6-well plates. After fixing with cold 100% methanol and staining with 0.5% crystal violet, cells were photographed using the Bio-Rad Gel Doc XR. The colony counts were measured by ImageJ analysis (NIH Bethesda, MD). The data represent the number of colonies ± standard error of the mean (SEM) of triplicates, and the experiments were repeated three times.

### Cell cycle analysis

RWPE-2, PC3, DU145, and MDA-PCa-2b cells were serum-starved overnight and transfected with NC or miR-214 mimic for 48 h and then harvested by trypsinization. Cells were washed in ice-cold phosphate-buffered saline (PBS) and fixed in ice-cold 70% ethanol overnight at −20 °C. Cell pellets were stained with FxCycl PI/RNase staining solution (Life Technologies Corporation) for 15 min at room temperature in the dark. Multicycle DNA cell cycle analyses were performed with a Cytoflex flow cytometer using FCS Express-6 software (De Novo Software, Glendale, CA). Data from 10,000 gated events/samples were collected for each data file, and the experiments were repeated three times.

### Apoptosis assay

The effect of miR-214 on cell apoptosis was determined by flow cytometry using an Annexin V-FITC and PI apoptosis detection kit (Life Technologies Corporation). Cells were transfected with miR-214 or NC mimic alone for 48 h or transfected cells further treated with Docetaxel (DTX, 1 nM) for an additional 24 h. Cells were washed in cold PBS, re-suspended in 1× annexin-binding buffer, and stained with Annexin V-FITC and PI. After 15 minutes of incubation at room temperature in the dark, cells were mixed with 1× annexin-binding buffer, kept on ice and analyzed for cell apoptosis using a Becton-Dickinson (BD) FACS Canto II flow cytometer (BD Biosciences).

### Western blotting

Western blotting was performed as described previously^71^. After transfection with miR-214 or NC mimic for 48 h, cell lysates were prepared from RWPE-2, PC3, DU145, MDA-PCa-2b, and LNCaP cells using lysis buffer (Cell Signaling Technology, Danvers, MA) containing a protease inhibitor cocktail (Roche, Indianapolis, IN). Cell lysates were also prepared from RWPE-2, PC3, DU145, and MDA-PCa-2b cells after transfection with empty vector or PTK6 plasmid for 24 h. Protein concentrations were determined using the Bio-Rad protein assay reagent (Bio-Rad, Hercules, CA). Cell lysates (40 µg) were separated on NuPAGE 4–12% Bis-Tris-SDS gels (Invitrogen) and then transferred to a polyvinylidene difluoride membrane (Millipore, Billerica, MA). The membrane was blocked in casein blocking buffer (1×) (Sigma-Aldrich, St. Louis, MO) and incubated with primary antibodies against cleaved PARP, PTK6, E-Cadherin, N-Cadherin, and GAPDH (Cell Signaling Technology) overnight at 4 °C. The following day, membranes were washed and subsequently incubated with the appropriate HRP-conjugated secondary antibodies for 1 h at room temperature. Following this incubation, membranes were washed and developed with an enhanced chemiluminescent detection system (Thermo Fisher Scientific, Waltham, MA).

### Migration assay

The wound healing migration assay was used to evaluate the effect of the miR-214 overexpression on the migratory ability of PCa cells. Cells were first transfected with miR-214 or NC mimic. Next, transfected cells were plated, and the cell monolayer was scraped using a micropipette tip (A_0_). At 24 h post-wounding (A_24_), cells were photographed, and the migration gap length was calculated using ImageJ software. The percent wound closure was calculated using the formula [(A_0_ – A_24_)/A_0_] × 100. All analyses were performed in duplicate with three independent experiments.

### Invasion and 3D spheroid assays

The effect of miR-214 overexpression on RWPE2 and PCa cell invasiveness was determined using Cultrex Basement Membrane Extract (BME) Cell Invasion Assay Kits and the 3D Spheroid BME Cell Invasion Assay (R&D Systems, Minneapolis, MN) following the manufacturer’s instructions. For the Matrigel invasion assay, miR-214- or NC-transfected PC3 cells were re-suspended in RPMI 1640 serum-free medium (1 × 10^5^ cells/well) and seeded onto the upper compartment of the chamber. This upper compartment was pre-coated with BME to form a matrix barrier in a 24-well plate. RPMI medium containing 5% FBS (0.6 ml), which acted as a chemoattractant, was added in the lower chamber. After 48 h, the invading cells present on the lower surface of the filter member were stained with Calcein-AM and incubated for 2 h at 37 °C in a cell culture incubator. Invading cell-associated fluorescence intensities were measured with a FluorStar Omega microplate reader using 485 nm excitation and 520 nm emission. The number of invading cells on the lower surface of the filter membrane was also observed and photographed using a Nikon TE-2000-E fluorescence microscope.

For the 3D spheroid assay, 3,000 transfected cells were re-suspended in 50 μl spheroid formation ECM solution at 24 h post-transfection, pelleted in 96-well round bottom plates, and then incubated at 37 °C for 72 h. On day 3, serum-supplemented invasion matrix was added to each sphere, and images were acquired using a Nikon TE-2000-E microscope. After incubating for 7 days at 37 °C, cell invasion was observed, and images were taken. The area of each spheroid was measured on days 3 (pre-invasion) and 10 (post-invasion) using ImageJ software (NIH). The difference was used to calculate the total area of cell invasion. Results are presented as the mean ± SEM from three independent experiments.

### Real-time quantitative PCR (RT/qPCR)

Total microRNAs from RWPE-2, PC3, DU145, MDA-PCa-2b, and LNCaP cells or cells transfected with NC or miR214 mimic were isolated using the mirVana microRNA Isolation Kit (Thermo Fisher Scientific). Total microRNAs (10 ng) were reverse transcribed using primers specific for miR-214 and U44 (Assay IDs 002306 and 001094, Applied Biosystems, Carlsbad, CA) and TaqMan Reverse Transcription reagents (Applied Biosystems). Expression of miR-214 and U44 was quantified by RT/qPCR using TaqMan PCR master mixture and Taqman expression assay primers, where U44 expression was used as an internal control.

The effects of miR-214 or NC mimic transfection on the expression of *PTK6* mRNA were analyzed by RT/qPCR. Briefly, total RNAs from RWPE-2, PC3, DU145, and MDA-PCa-2b cells were isolated using TRIZOL Reagent (Thermo Fisher Scientific). RNA (1 µg) was reverse transcribed using a High Capacity cDNA Reverse Transcription kit (Applied Biosystems). cDNA was incubated with Power SYBR Green PCR master mix (Applied Biosystems) and *PTK6* forward 5′-CCTCTCCCATGACCACAATATC-3′ and *PTK6* reverse 5′- GAGAATCCCAAAGGACCAGAC-3′ primers. *GAPDH* was amplified as an internal control using *GAPDH* forward 5′-CCACCCAGAAGACTGTGGAT-3′ and *GAPDH* reverse 5′-GTTGAAGTCAGAGGAGACCACC -3′ primers. The PCR mixtures were run on a QuantStudio-3 PCR System (Applied Biosystems) using relative quantitation according to the manufacturer’s protocols.

### Luciferase assay

PC3 cells were seeded onto 96-well plates and transfected with 1 µg of PTK6-3′UTR-Luciferase reporter construct for 18 h. Cells were then transfected with 50 nM miR-214 (wild-type), or miR-214 (mutant) mimic or NC mimic for an additional 24 h. Transfected cells were lysed, and luciferase activity was measured using the light switch assay system (Switchgear Genomics, Carlsbad, CA) following the manufacturer’s instructions.

### Immunofluorescence

For immunostaining, PC3 cells (1.5 × 10^5^) were grown on coverslips in 6-well plates and transfected with custom Cy3-labeled NC or miR-214 mimic (Bioneer, Oakland, CA) for 48 h. Cells were washed with PBS, fixed with 4% paraformaldehyde for 15 min, then cells were blocked with 2% bovine serum albumin for 1 h and incubated with anti-N-Cadherin, and anti-E-Cadherin rabbit primary antibodies (diluted 1:200) at 4 °C for 18 h. For PTK6 staining, transfected cells were fixed and permeabilized with 0.25% Triton X-100 for 15 min and cells were blocked and incubated with anti-PTK6 rabbit primary antibody (diluted 1:200) at 4 °C for 18 h. Cells were washed twice with PBS and incubated with Alexa-Fluor® 488-conjugated fluorescein-labeled anti-rabbit secondary antibody (Invitrogen). Following immunostaining, cells were mounted with Vectashield mounting medium (Vector Laboratories, Burlingame, CA) containing a nuclear DAPI (4′,6-diamidino-2-phenylindole) stain. Images were captured by confocal microscopy using a ZEISS LS 800 microscope.

### *In vitro* kinase assay

The effect of ibrutinib on kinase inhibition was performed using an *in vitro* kinase assay (Luceome Biotechnology, Tucson, AZ). For each kinase assay, Cfluc-Kinase and Fos-Nfluc were translated using a cell-free system (rabbit reticulocyte lysate) at 30 °C for 90 min. The lysates (duplicates) were first incubated with DMSO or 1 µM ibrutinib for 30 min at room temperature and then incubated with a kinase-specific probe for another hour. After incubation, luciferase assay reagent (80 µl) was added, and luciferase activity was measured using a Luminometer. The percentages of kinase inhibition were determined as described previously^47^.

### Statistical analysis

Results are presented as mean ± SEM of 3 independent experiments, each performed in triplicate unless noted otherwise. Statistical significance between means was determined by Graph Pad Prism 8 software (GraphPad Software Incorporated, La Jolla, CA) using one-way or two-way ANOVAs with Tukey’s multiple comparisons test or multiple t-tests (statistical significance determined using the Holm-Sidak method) when appropriate. Differences were considered significant at *P* < 0.05, and *P* values are shown in the figures.

## Supplementary information

MicroRNA-214 targets PTK6 to inhibit tumorigenic potential and increase drug sensitivity of prostate cancer cells

## Data Availability

All data produced during the current study are included in this article and its Supplementary Files.
