# Supplementary material for: MicroRNA-214 targets PTK6 to inhibit tumorigenic potential and increase drug sensitivity of prostate cancer cells
Source: Sci Rep. 2019 Jul 5;9:9776. doi: 10.1038/s41598-019-46170-3 (PMC6611815; doi:10.1038/s41598-019-46170-3)
Supplement: Supplementary file 1 — MicroRNA-214 targets PTK6 to inhibit tumorigenic potential and increase drug sensitivity of prostate cancer cells [file 41598_2019_46170_MOESM1_ESM.pdf]

MicroRNA-214 targets PTK6 to inhibit tumorigenic potential and increase drug sensitivity of prostate cancer cells

Patrice Cagle[1], Suryakant Niture[1], Anvesha Srivastava[2], Malathi Ramalinga[2], Rasha Aqeel[2], Leslimar Rios-Colon[1], Uchechukwu Chimeh[1], Simeng Suy[4], Sean P. Collins[4], Rajvir Dahiya[5] and Deepak Kumar[1,2,3*]

[1]Julius L. Chambers Biomedical Biotechnology Research Institute, North Carolina Central University, Durham, NC 27707
[2]Cancer Research Laboratory, Division of Science and Mathematics, University of the District of Columbia, Washington, DC 20008
[3]Department of Pharmaceutical Sciences, North Carolina Central University, Durham, NC 27707
[4]Department of Radiation Medicine, Georgetown University, Washington, DC 20057
[5]VA Medical Center and University of California San Francisco CA 94121

Supplementary material

S2.C

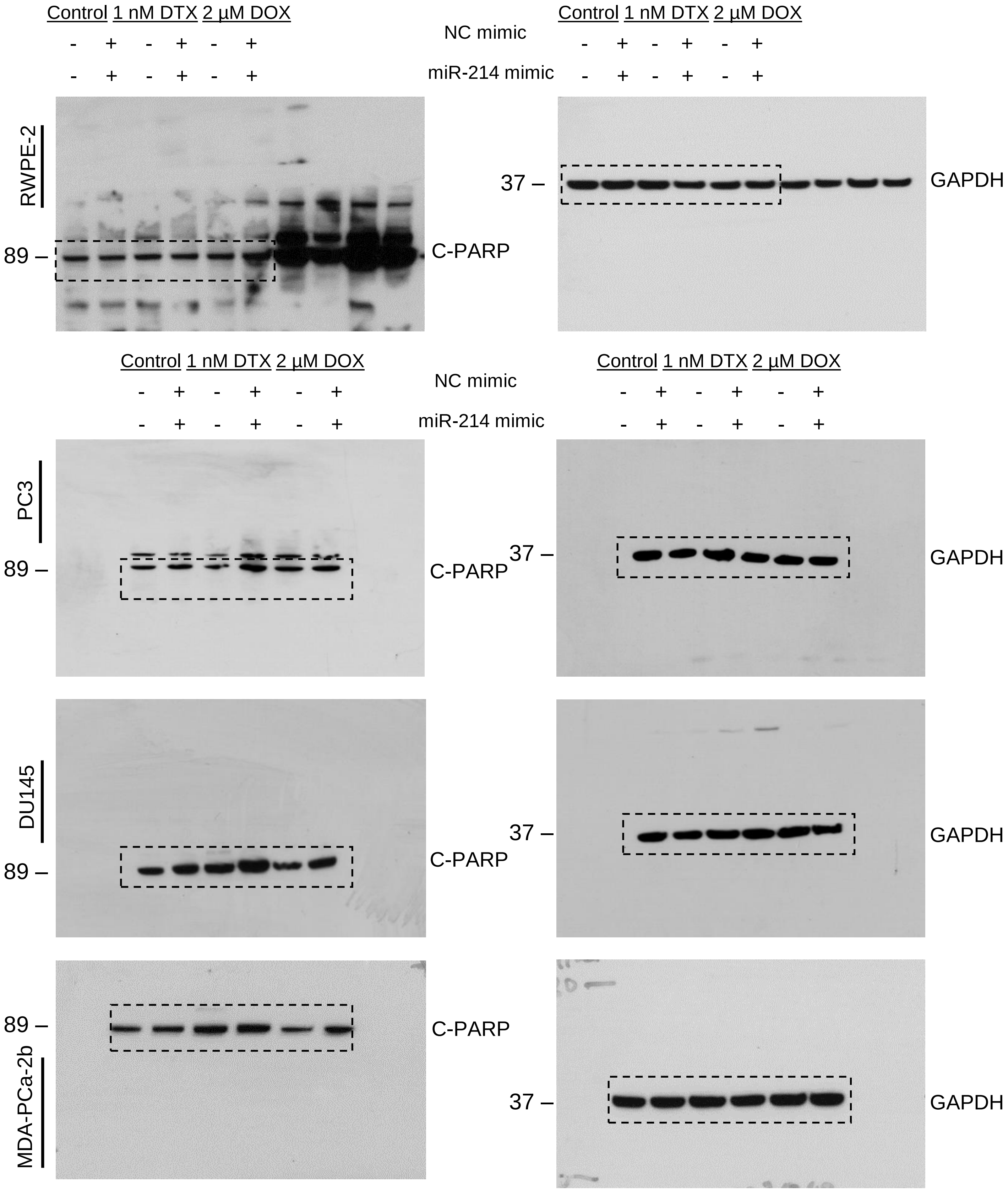

Supplementary figure 2.C (S2.C): Full length images of blots for Fig 2 C in main paper. Dotted rectangles indicate the regions used in the figures. PVDF membranes were used for probing with the respective primary antibody.

S3.E

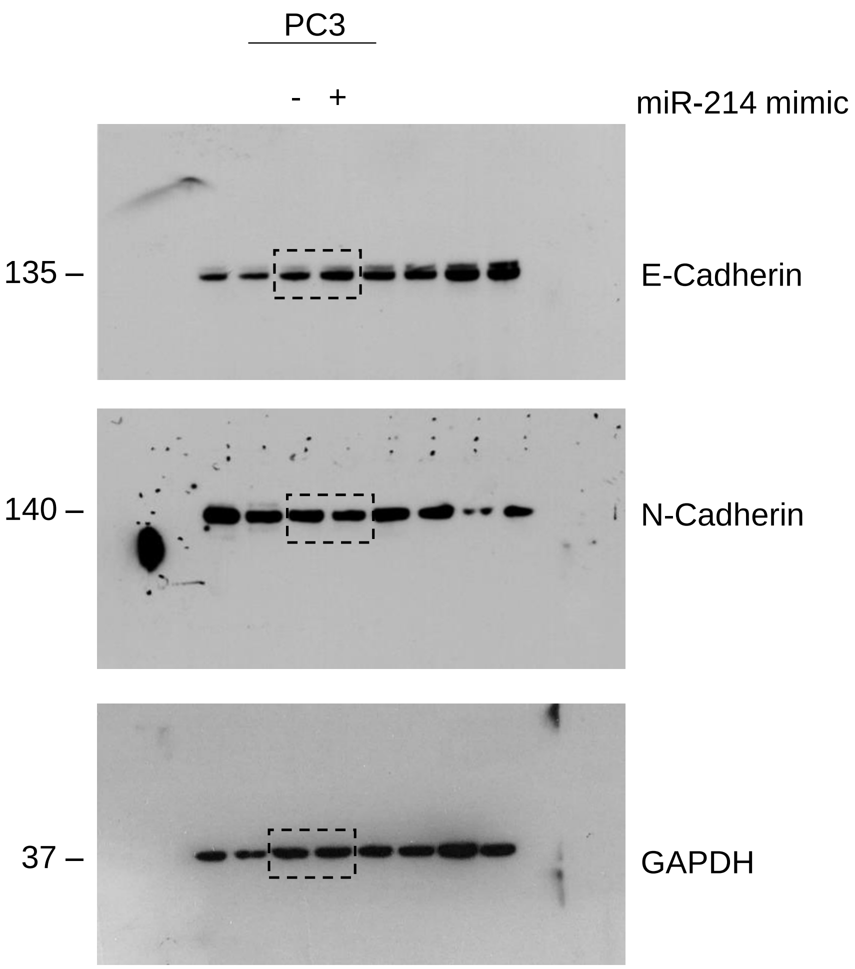

Supplementary figure 3.E (S3.E): Full length images of blots for Fig 3 E in main paper. Dotted rectangles indicate the regions used in the figures. PVDF membranes were used for probing with the respective primary antibody.

S4.D

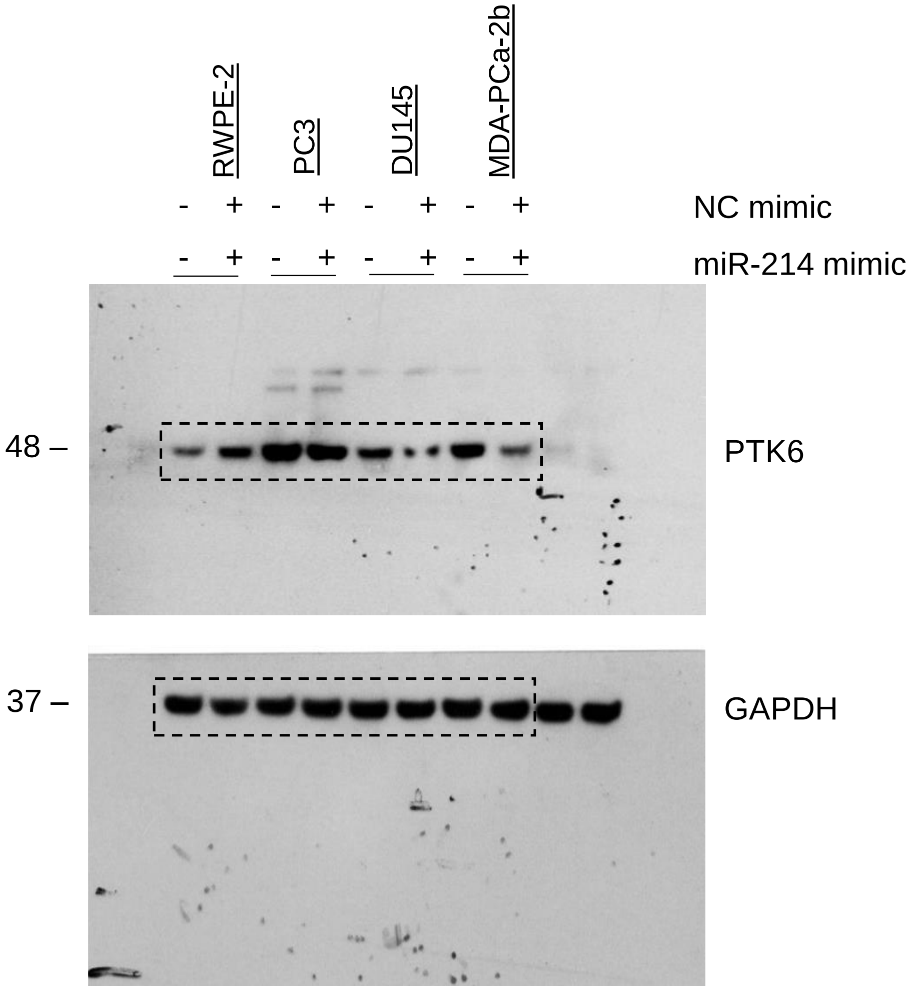

Supplementary figure 4.D (S4.D): Full length images of blots for Fig 4 D in main paper. Dotted rectangles indicate the regions used in the figures. PVDF membranes were used for probing with the respective primary antibody.

S4.F

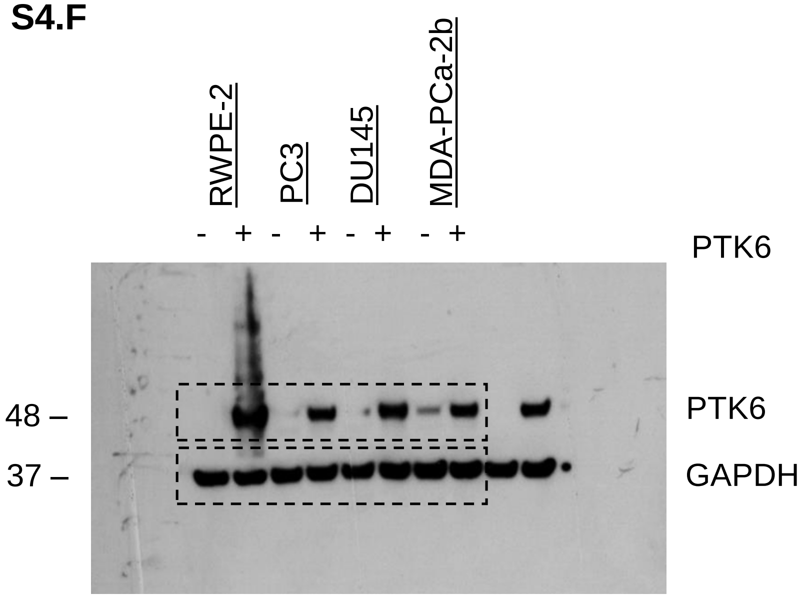

Supplementary figure 4.F (S4.F): Full length images of blots for Fig 4 F in main paper. Dotted rectangles indicate the regions used in the figures. PVDF membranes were used for probing with the respective primary antibody.

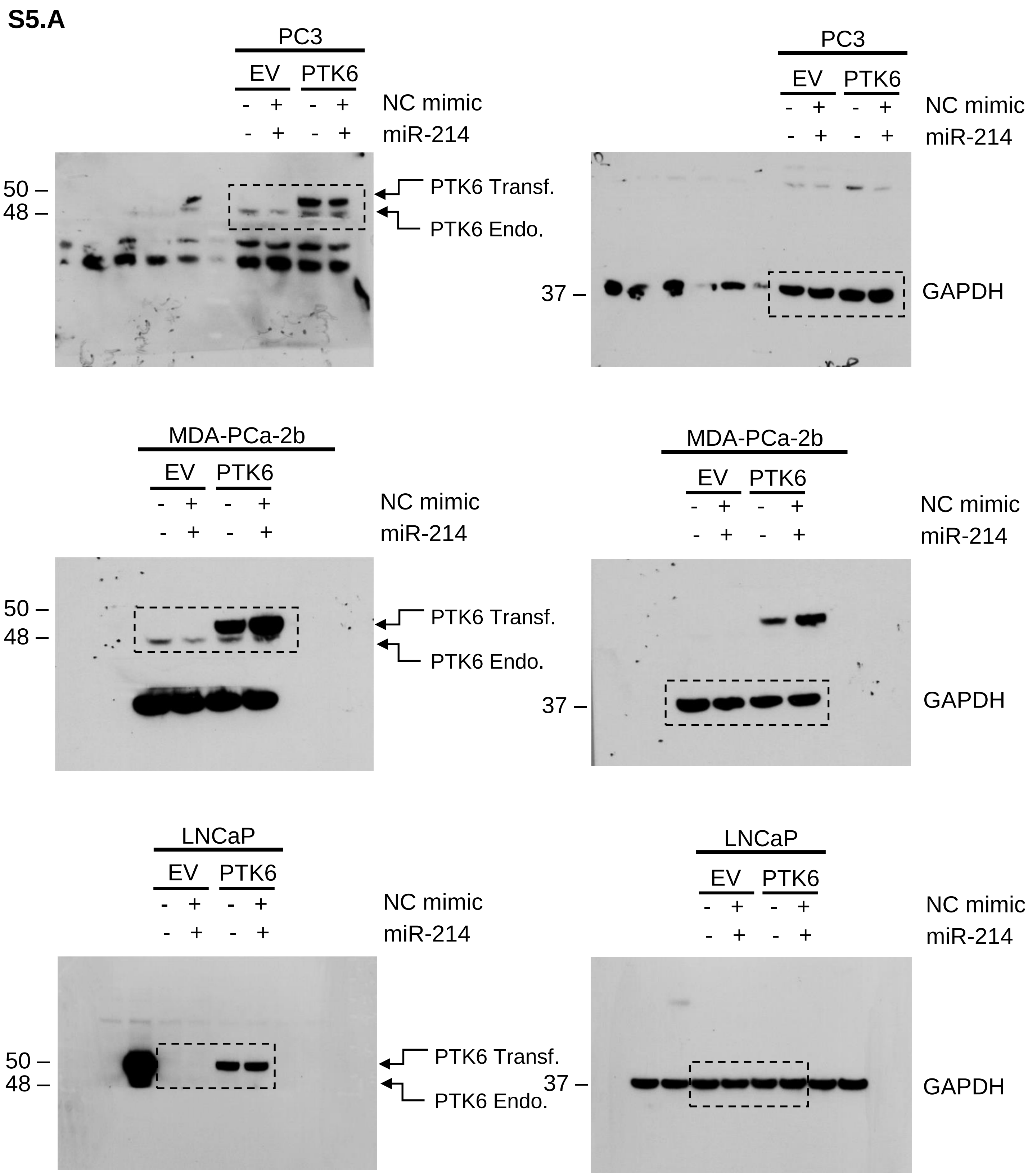

Supplementary figure 5.A (S5.A): Full length images of blots for Fig 5 A in main paper. Dotted rectangles indicate the regions used in the figures. PVDF membranes were used for probing with the respective primary antibody.
